# Emerging trends in the management of difficult-to-treat rheumatoid arthritis: targeted treatments and non-pharmacological interventions

**DOI:** 10.1093/rheumatology/keag217

**Published:** 2026-04-22

**Authors:** György Nagy, Koshiro Sonomoto, András M Dorgó, Lilla Gunkl-Tóth, Yoshiya Tanaka

**Affiliations:** Department of Rheumatology and Immunology, Semmelweis University, Budapest, Hungary; Heart and Vascular Center, Semmelweis University, Budapest, Hungary; Department of Genetics, Cell- and Immunobiology, Semmelweis University, Budapest, Hungary; Department of Clinical Nursing, School of Health Sciences, University of Occupational and Environmental Health, Japan, Kitakyushu, Japan; Department of Rheumatology and Immunology, Semmelweis University, Budapest, Hungary; Department of Rheumatology and Immunology, Semmelweis University, Budapest, Hungary; Department of Pharmacology and Pharmacotherapy, University of Pécs, Pécs, Hungary; Chronic Pain Research Group, Hungarian Research Network – University of Pécs (HUN-REN – PTE), Pécs, Hungary; Department of Molecular Targeted Therapeutics, University of Occupational and Environmental Health, Japan, Kitakyushu, Japan; The First Department of Internal Medicine, School of Medicine, University of Occupational and Environmental Health, Japan, Kitakyushu, Japan

**Keywords:** difficult-to-treat, D2T, rheumatoid arthritis, b/tsDMARDs, JAK inhibitors, non-pharmacological

## Abstract

Patients with RA often require alterations of their therapeutic regimen throughout the course of their illness, guided by specific outcomes in line with the treat-to-target principle. Importantly, a significant portion of the RA population fails to achieve sufficient disease control in the long term. Several years ago, the umbrella notion of difficult-to-treat (D2T) RA was introduced to facilitate more structured care and research efforts for patients who remain symptomatic despite having received multiple courses of targeted medications. D2T RA represents a heterogeneous condition sustained by a complex interplay of underlying factors. Biological therapies and Janus kinase inhibitors have demonstrated efficacy in alleviating disease activity in patients refractory to conventional treatments. The use of tailored non-pharmacological therapies (physiotherapy, psychological interventions, etc.) to complement pharmacotherapy chiefly addresses symptoms unrelated to ongoing inflammation. In this narrative review, we examine the current use and relevance of pharmacological and non-pharmacological approaches for D2T RA.

Rheumatology key messages

- Treatment alterations may result in adequate disease control for some patients with D2T RA.
- Emerging real-world evidence suggests JAK inhibitors may be particularly effective in D2T populations.
- Non-pharmacological approaches should be considered for all patients with RA, especially in non-inflammatory D2T disease.

## Introduction

RA is a systemic autoimmune disease primarily affecting the synovial tissue that often leads to widespread pain and persistent physical dysfunction. Symptoms often improve with adequate disease control, although irreversible physical impairment associated with bone and cartilage destruction may arise [1, 2]. Additionally, the condition may involve extra-articular sites, potentially contributing to an unfavourable prognosis. RA carries a substantial disease burden globally, with the number of patients steadily increasing over the past decades [3].

To control the progression of joint damage and improve prognosis, therapeutic strategies have continued to evolve over the past 30 years [4]. Guidelines prioritize early diagnosis and treatment of RA and optimal care based on shared decision-making, with the ultimate goal of improving long-term outcomes. Methotrexate (MTX), a conventional synthetic (cs) DMARD, is recommended as first-line therapy when diagnosis is established and no contraindications exist [5–7]. If the target is not reached within six months of MTX use, a biological (b) DMARD or a targeted synthetic (ts) DMARD, i.e. a Janus kinase (JAK) inhibitor, should be added. If the goal is still not met within the following 3–6 months, switching to another b/tsDMARD is recommended.

The treat-to-target (T2T) strategy provides a practical framework for the establishment of clear treatment targets and regular adjustments to the therapeutic plan to achieve and maintain these goals [8]. The main objectives are to achieve remission or, alternatively, low disease activity (LDA), and to prevent progression of structural damage and physical dysfunction. It is recommended to adjust drug therapy every 3–6 months until the desired goals are achieved. Real-world evidence shows that the implementation of the T2T strategy improves remission rates, not only by preventing structural damage but also by contributing to pain control and improvement of physical function and health-related quality of life (QoL) [9]. These findings emphasize that actively guiding RA to remission leads to the prevention of long-term disabilities and delivers true clinical benefits. However, a disparity between physicians’ perceptions and the reality of T2T implementation may occur [10]. Furthermore, utilizing multiple targeted therapies does not always provide sustained symptom control despite a T2T approach, leaving both patients and healthcare providers with an ongoing challenge in reaching the predefined target [11].

The current narrative review focuses on patients who continue to experience difficult-to-treat (D2T) RA despite appropriate care. Our main aim was to summarize the key findings and insights into the current use, potential benefits, and safety profile of b/tsDMARDs and non-pharmacological/non-surgical interventions in patients with D2T RA (and comparable populations). We conducted a literature search in PubMed and Scopus to identify relevant papers (published up to March 2026). Keywords included ‘difficult-to-treat’, ‘rheumatoid arthritis’, ‘b/tsDMARDs’, ‘non-pharmacological’, and related terms. Additional papers were included to provide a wider clinical context for the discussion of various treatment approaches.

## The difficult-to-treat framework in RA

**Figure 1:**
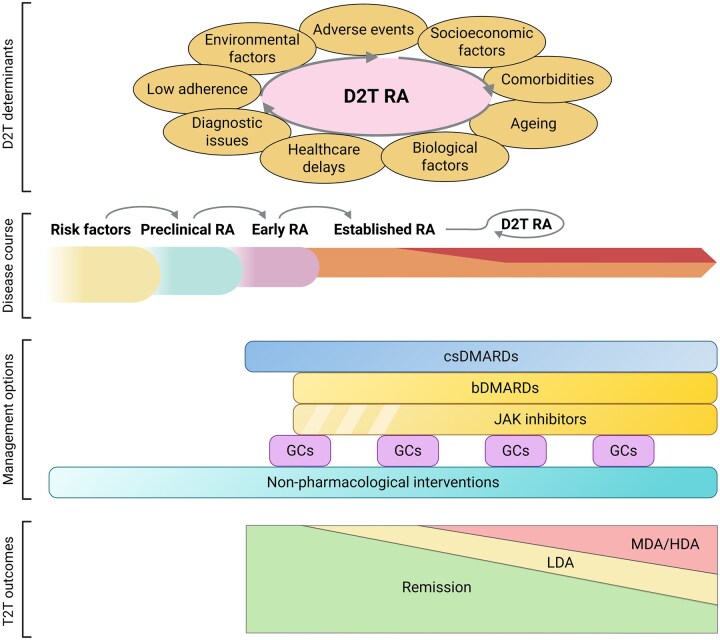
Disease states in RA with corresponding therapeutic approaches. In some patients with established RA, a D2T state may occur after the failure of reaching treatment targets (i.e. remission or at least LDA) despite cycling through several classes of targeted therapies (bDMARDs and JAK inhibitors) in line with the T2T strategy. However, subsequent treatment changes (e.g. escalation of DMARDs, addition of non-pharmacological interventions) may improve outcomes and help the patient transition out of this state. A combination of underlying factors may facilitate and maintain D2T RA, as also reflected by the clinical and biological heterogeneity of this patient group. Some of these determinants may already be evident before the development of the D2T state, underscoring the importance of thorough evaluation and timely intervention throughout the disease course. b: biological; cs: conventional synthetic; D2T: difficult-to-treat; GC: glucocorticoid; HDA: high disease activity; JAK: Janus kinase; LDA: low disease activity; MDA: moderate disease activity; T2T: treat-to-target. Figure 1 was created in BioRender: Tóth, L. (2026) https://biorender.com/btu4g14, accessed on 16 April 2026

A European Alliance of Associations for Rheumatology (EULAR) task force, established in 2018, developed a consensus-based terminology and definition for D2T RA (Fig. 1), with the aim of fostering more focused research into the underlying factors and optimal treatment approaches of this disease state. Under the uniform definition, three conditions are required for classification as D2T RA: (1) prior failure of at least two courses of b/tsDMARD treatment with different mechanisms of action (MoAs); (2) presence of signs suggesting active or progressive disease; and (3) a subjective assessment—by the patient and/or the physician—reflecting a difficulty in disease management [12]. A recent meta-analysis indicated a global prevalence of 10.9% for D2T disease in overall RA cohorts [13]. The D2T framework has since gained ground in other immune-mediated diseases as well [14].

In RA, a combination of different factors may contribute to consecutive treatment failures, and symptoms of patients are associated with varying levels of inflammation, explaining the clinical heterogeneity among D2T RA patients [15]. Systemic involvement and comorbidities complicate RA management in multiple ways, e.g. by limiting therapeutic options, heightening inflammatory activity, contributing to polypharmacy, or confounding assessment of disease activity. Furthermore, co-existing conditions may be elicited or exacerbated by RA or immunosuppressive treatments. This interplay makes them key players in the development and persistence of D2T disease, underscoring the importance of integrated care among medical specialties [16]. Comorbidities were found to be more common in D2T RA compared with non-D2T RA across multiple cohorts [17].

The effect of certain D2T contributors is amplified in the elderly population, posing an increasing challenge in the management of RA [18, 19]. Advancing age is accompanied with a higher comorbidity burden (e.g. osteoporosis, cardiovascular disease [CVD], poor mental health), frailty, and polypharmacy [20], which complicate disease assessment and therapeutic decisions. A Korean cohort study of elderly patients with RA reported an association between higher RAPID3 scores and D2T disease, and while survival rates of b/tsDMARDs were similar between D2T and non-D2T groups, inefficacy-related withdrawal was more frequent in D2T RA [21].

Non-adherent behaviour is a possible contributor to D2T RA, with the presence of adverse events identified as the most important barrier to treatment adherence [22]. Disparities in the healthcare trajectory of patients, including delays in care and diagnosis, as well as limited access to medication, may also affect RA outcomes [23–25]. The failure to start MTX within three months after diagnosis was found to be a predictor of D2T RA [26], and in a Dutch cohort, lower socioeconomic status at RA onset was identified as an independent risk factor for developing D2T disease [27]. Importantly, the D2T phenotype can also be driven by pain that persists despite adequate inflammation control [28]. Several factors may contribute to sustained pain in RA, such as pain syndromes (e.g. fibromyalgia), secondary osteoarthritis, as well as psychological comorbidities, which also influence pain perception.

Remarkably, some patients present signs of ongoing inflammation without evident underlying causes despite cycling through several courses of b/tsDMARDs. This suggests the existence of a disease phenotype mediated by poorly understood immunological pathways that remain untargeted by currently available medications, sometimes termed ‘true’ refractory RA [28].

The D2T designation does not presume a definitive disease status—individuals who meet the criteria at a certain point may shift back to non-D2T RA over time, especially if a revised treatment plan proves beneficial [29, 30]. The EULAR task force proposed points to consider (PtC) and a step-by-step algorithm for the management of D2T RA based on existing evidence [31], which also emphasize the implications of possible misdiagnosis and comorbidities.

Furthermore, the PtC highlight the importance of evaluating the presence of inflammation (e.g. using composite activity scores or clinical assessment) to guide therapeutic choices. In case of clinical doubt, musculoskeletal ultrasound has been proposed as a useful method for detecting inflammation [32, 33], and recent studies have also indicated that demonstrating objective evidence of synovitis on imaging [34–36] or histological samples [37] might lead to enhanced identification of the persistent inflammatory refractory phenotype [28]. Further escalation of DMARD therapy is generally considered beneficial primarily in the presence of heightened inflammatory activity, although a recent study indicated that some patients with low-inflammation D2T RA may also benefit from therapeutic switching [38]. On the other hand, the addition of non-pharmacological treatment approaches should be considered for all patients, particularly when the main symptoms do not appear to be driven by inflammation [31, 39].

## Current targeted treatment through the D2T lens

The introduction of targeted therapies has significantly expanded the therapeutic landscape in RA by offering novel modes of action that directly intervene in dysregulated immune pathways, enabling management of more complex cases. However, managing patients who fail to achieve optimal responses remains a major challenge in clinical practice. It is estimated that symptoms are driven by persistent inflammatory activity in nearly half of patients with D2T disease [13], for whom appropriate selection of b/tsDMARDs is a critical management step. Multidrug resistance remains an important unmet need in the treatment of RA, which may be partly ascribed to a trial-and-error approach due to the lack of robust predictive markers of therapeutic response and clear, evidence-based guidance on optimal treatment sequencing. This therapeutic gap highlights the need not only for the development of new treatment options but also for more precise use of available medications to potentially address the diversity in the biological profiles of patients with D2T RA.

### Biological treatments

Tumour necrosis factor (TNF) inhibitors were the first biological drugs to be approved for the management of RA, and in most real-world cohorts, they remain the most common initial choice of targeted treatment following csDMARD failure. Tocilizumab and sarilumab are interleukin (IL)-6 receptor inhibitors used for the treatment of RA. The RADIATE trial confirmed the efficacy of tocilizumab in patients with RA refractory to TNF inhibitors [40], and stratified analyses found that intravenous tocilizumab at both 4 mg/kg and 8 mg/kg exhibited superior American College of Rheumatology (ACR) 20% responses compared with placebo, with similar response rates among patients with a history of one, two, or three previous failures of anti-TNF therapy [40]. In a Japanese observational study of 150 RA patients, the use of IL-6 receptor inhibitors was associated with resolution of the D2T status [29].

The fusion protein abatacept blocks the co-stimulation of T cells. In a head-to-head trial comparing abatacept and the JAK inhibitor upadacitinib in bDMARD-refractory patients, the upadacitinib-treated group demonstrated greater improvement in disease activity and higher rates of remission at week 12, although this was accompanied by more serious adverse events [41]. A subgroup analysis of patients with a bDMARD history of ≥2 MoAs or ≥3 individual agents indicated a similar result in the change of disease activity [41]. Rituximab, a CD20 inhibitor, has been associated with favourable rates of clinical response in real-world D2T RA cohorts [42, 43].

### JAK inhibitors

JAK inhibitors have the potential to maintain efficacy even in cases of inadequate response (IR) to multiple prior bDMARDs. The SELECT-BEYOND trial of 499 patients with IR to bDMARDs compared a group receiving upadacitinib 15 mg once daily and a placebo group, with csDMARDs as background therapy [44]. At week 12, the ACR20 response rate was 64.6% in the upadacitinib 15 mg group, significantly higher than 28.4% in the placebo group, meeting the primary endpoints. This drug demonstrated a similar ACR20 improvement rate in cases of IR to ≥3 bDMARDs with the same or ≥2 drugs with different MoAs, as well as in cases of IR to anti-IL-6 agents. The FINCH 2 trial was a comparative study of filgotinib 200 mg once daily in combination with csDMARDs vs placebo in 448 patients with IR to bDMARDs [45]. At week 12, the ACR20 response rate was 66.0% in the filgotinib 200 mg group, significantly higher than the 31.1% in the placebo group, meeting the primary end point. This drug also demonstrated similar efficacy in a subgroup with IR to ≥3 bDMARDs. In the RA-BEACON trial, which compared 4 mg once-daily baricitinib plus csDMARD with a placebo group in patients with IR to TNF inhibitors, the ACR20 improvement rate at 12 weeks was 55% with 4 mg baricitinib, significantly higher than 27% in the placebo group. In a subgroup analysis, baricitinib outperformed placebo in patients with IR to ≥3 bDMARDs [46]. Nevertheless, these results are merely descriptive subgroup analyses, and relevance can only be confirmed through direct comparative trials in D2T RA patients.

In a single-centre study in France that compared 45 D2T and 29 non-D2T RA patients, JAK inhibitors significantly reduced DAS28-CRP in both groups, with no clear difference in efficacy [47]. However, in an observational study of 159 patients in Japan, the retention rate of JAK inhibitors was significantly lower in the D2T RA group than in the non-D2T RA group (64.0% vs 78.4% at 6 months), and the rate of Clinical Disease Activity Index (CDAI)-defined LDA at 6 months was also lower in the D2T group (34%) compared with non-D2T patients (62.3%) [48].

### Considerations for D2T disease

Multiple studies suggest that repeated b/tsDMARD failures tend to be associated with worse outcomes and an increased risk of further therapeutic failures [49–51]. A Japanese observational study of 330 patients found that, one year after starting a new targeted therapy, the success rate was 31% in D2T disease vs 63% in the non-D2T group [52]. When stratified by MoA, JAK inhibitors and abatacept showed significantly lower success rates in the D2T group, and TNF inhibitors and anti-IL-6 treatment also exhibited reduced rates (although not significantly) [52]. Nonetheless, D2T and non-D2T groups did not show significant differences in b/tsDMARD retention rates in a Korean nationwide cohort, although discontinuation due to inefficacy was more frequent in D2T RA [53]. In studies from our multicentre cohort in Japan, the FIRST Registry, past failure of ≥4 b/tsDMARDs was identified as an independent cut-off value associated with poor response to a subsequent targeted agent [49], and JAK inhibitors led to the greatest clinical improvements in D2T groups with prior b/tsDMARD failure of ≥2 as well as ≥3 MoAs [54].

The EULAR PtC indicate that switching between classes might be beneficial after failure of a second (or further) b/tsDMARD, particularly after two TNF inhibitors [31]. Previously, a systematic review found that non-TNF inhibitor biologics tend to show higher efficacy than TNF inhibitors in patients who have previously failed at least one anti-TNF agent [51]. This corresponds with findings from a network meta-analysis that, after failure of a first-line TNF inhibitor, switching to a drug with a different MoA is more effective and results in a lower risk of discontinuation when compared with anti-TNF cycling [55]. The JAK-pot collaboration explored the value of switching after failure of the first JAK inhibitor, concluding that cycling to a second JAK inhibitor and switching to a biological agent resulted in similar retention rates at 2 years [56]. A recent study found that 51.1% and 42.9% of patients who initiated a third or fourth new MoA, respectively, achieved treatment targets, supporting the usefulness of between-class switching in D2T RA [25].

Real-world data have also provided insights into the outcomes of different targeted drug classes in D2T RA. In a Spanish retrospective study of 122 patients who initiated a new b/tsDMARD after meeting D2T RA criteria, 75 patients (61.5%) maintained treatment. Among different classes, rituximab showed the highest survival, followed by JAK inhibitors and IL-6 receptor inhibitors, whereas abatacept and TNF inhibitors had lower survival rates [42]. In a Japanese cohort, 826 treatment courses of 450 D2T RA patients were evaluated, and discontinuation rates due to inefficacy were lower for JAK inhibitors and anti-IL-6 receptor antibodies compared with TNF inhibitors and abatacept, although CDAI scores showed similar trajectories over one year across the four treatment groups [57]. A single-centre study of 66 patients meeting D2T RA criteria found that patients achieved remission or LDA most frequently with rituximab (69%) and JAK inhibitors (64%), with lower rates for IL-6 receptor inhibitors (41%) and no improved cases with abatacept and TNF inhibitors [43]. In the FIRST Registry, 353 patients with D2T RA received treatment: 71 with TNF inhibitors, 79 with IL-6 receptor inhibitors, 58 with abatacept, and 145 with JAK inhibitors. Multivariate analysis revealed that CDAI improvement at 1 year after initiation was equivalent to that of TNF inhibitors for IL-6 receptor inhibitors and abatacept, whereas JAK inhibitors demonstrated a significantly greater improvement compared with TNF inhibitors. The hazard ratios for adverse events leading to discontinuation were 0.64 for IL-6 receptor inhibitors, 0.45 for abatacept, and 0.37 for JAK inhibitors compared with TNF inhibitors, with no significant differences among the four groups [54]. Nevertheless, it must be recognized that observational studies often face limitations in clearly demonstrating potential confounding by indication.

As many patients with D2T disease experience ongoing pain despite adequate control of inflammation, understanding the analgesic potential of various targeted treatments is also pertinent in this context. In a low-inflammation D2T RA cohort from the FIRST Registry, patients who switched between b/tsDMARDs achieved greater improvements in CDAI and pain compared with the non-switch group [38], suggesting that treatment escalation may be beneficial even in the absence of high inflammation. Clinical data suggest that JAK inhibitors may be more effective than biologics in improving patient-reported outcomes (PROs) such as pain and fatigue in RA [58, 59]. Several cytokines involved in RA pathogenesis are also implicated in pain processing, some of which (e.g. IL-6 and interferons) signal directly through the JAK/STAT pathway, whereas others (e.g. IL-1 and TNF) modulate related routes indirectly. This overlap offers a plausible mechanistic link between JAK inhibition and pain-related pathways, including mechanisms of peripheral and central sensitization [60, 61], pointing to potential benefits in D2T RA complicated by non-inflammatory pain. Preliminary findings from ongoing functional magnetic resonance imaging studies in RA suggest that JAK inhibitors may influence nociplastic pain features in the brain [62, 63], although these observations remain exploratory. Concurrently, multiple studies indicate that TNF blockade may also affect central pain processes [64–66], suggesting that such actions are not specific to JAK inhibition. Current evidence remains insufficient to draw firm conclusions; nevertheless, these findings suggest a potential role for targeted therapies in pain mechanisms, warranting further investigation.

The combination of targeted therapies with a csDMARD leads to better clinical response in RA. However, recent findings indicate lower proportions of concomitant csDMARD use [27, 53, 67] and an increased rate of MTX contraindication/intolerance in the D2T population [25]. A Japanese study found a significantly higher MTX dose among those who responded favourably to a targeted treatment in D2T RA, with a weekly dose of ≥8.7 mg defined as cut-off for therapeutic success [52]. Interestingly, in the FIRST Registry, the superiority of JAK inhibitors over other classes with respect to CDAI response in D2T disease was more apparent among patients with no concomitant MTX or glucocorticoid use [54]. Overall, the use of csDMARDs may offer added benefits even in D2T disease, calling for investigation into optimal combination regimens.

Beyond efficacy, the choice of b/tsDMARD in RA should also consider the safety profile of available agents. The use of targeted therapies carries risks of adverse events; for instance, a meta-analysis showed an increased risk of severe infections in patients with RA using biologics compared with csDMARDs [68]. In patients with comorbidities, the priority of certain drugs may be reduced [69], and various guidelines include warnings about the use of targeted therapy in patients with potential infections or concerns about malignant tumours [70, 71]. For example, in patients with a history of malignancy, JAK inhibitors and abatacept should be administered with caution only when no other options are available [72]. In the ORAL Surveillance trial, which enrolled patients aged ≥50 years with cardiovascular risk, tofacitinib did not demonstrate non-inferiority to TNF inhibitors for the incidence of malignant tumours and major adverse cardiovascular events [73]. *Post hoc* analysis indicated that several characteristics (e.g. ≥65 years of age or current/past smoking) were associated with higher risk, suggesting the possibility of risk stratification [74, 75]. Observational studies do not clearly indicate an increased incidence of malignancies among patients treated with JAK inhibitors, including tofacitinib [76]. Nevertheless, the latest expert consensus statement emphasizes the importance of thoroughly considering the potential benefits and risks when using JAK inhibitors, alongside pretreatment screening and regular safety monitoring [70]. In our FIRST Registry, plain CT screening prior to b/tsDMARD administration—in addition to more general risk assessment—has allowed early detection of potential malignant tumours and infections, leading to more favourable clinical outcomes [77–79].

Comprehensive screening efforts are expected to contribute to reducing treatment challenges in RA by identifying and addressing potential risks in advance, thereby enabling the safe and appropriate use of targeted therapies. However, evidence suggests that improvements in physical function remain limited despite targeted therapy, particularly in patients aged ≥75 years [80]. In many countries, the treatment of RA is largely confined to drug therapy, and comprehensive management including non-pharmacological interventions has been difficult to implement. These results highlight the importance of multimodal management combining diverse intervention methods, as drug therapy alone is insufficient in this context.

## Non-pharmacological approaches to D2T RA

While RA management chiefly relies on the application of different pharmacological modalities, DMARDs and glucocorticoids may be less effective in addressing certain complaints, especially those not directly related to inflammatory activity. The EULAR PtC for the management of D2T RA underline the potential value of non-pharmacological options in mitigating symptoms such as pain, fatigue, and functional impairment [31]. It has been suggested that the additional benefit of these interventions may be even larger in D2T RA, yet formal evidence in this specific subgroup remains scarce [51]. Hence, their application in D2T disease can be justified by extrapolating findings from studies in more general RA populations [39] and by relevant guidelines for their use in inflammatory arthritis [6, 81–84]. The generally favourable safety record of non-pharmacological therapies also adds to their potential value in the D2T population typically burdened by comorbidities and drug-related adverse events. Remarkably, the ACR has published a guideline that includes a broad range of integrative (non-pharmacological) interventions in RA [85], although most of these recommendations are conditional due to limited evidence.

**Figure 2:**
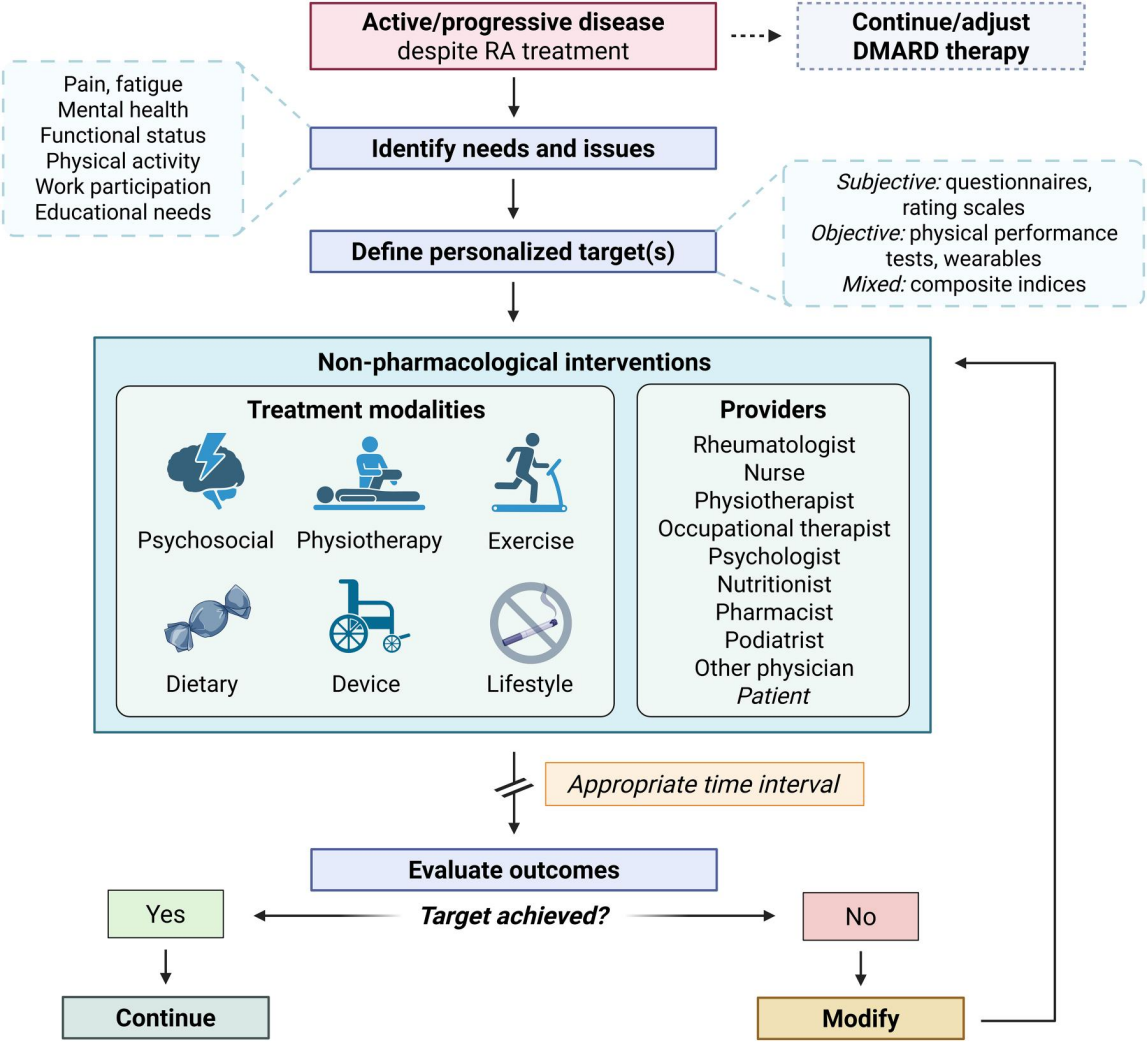
Individualized algorithm for the selection and delivery of non-pharmacological/non-surgical therapies in D2T RA. Optimal care for D2T RA requires a holistic management approach that extends beyond pharmacotherapy. In this setting, a comprehensive assessment of key functional domains (e.g. pain, fatigue, functional status) may uncover unmet patient needs and problems that can be addressed by non-pharmacological interventions. This evaluation allows for the establishment of personalized therapeutic targets using subjective or objective outcome measures. At the same time, a ‘package of care’ consisting of one or more treatment modalities can be planned and implemented by members of the multidisciplinary team (rheumatologist, psychologist, physiotherapist, etc.). The selection of goals and interventions should occur in the context of shared decision-making between the patient and healthcare providers. Over time, repeated assessment of the previously defined targets can help monitor the effectiveness of therapy and inform subsequent management decisions. D2T: difficult-to-treat. Figure 2 was created in BioRender: Tóth, L. (2026) https://biorender.com/zclk4l6, accessed on 16 April 2026

When introducing or expanding the multimodal ‘package of care’ for patients with ongoing symptoms despite pharmacological treatment, therapeutic choices should be based on a shared decision between the patient and healthcare providers, akin to the application of drug therapy within the T2T framework [86] (Fig. 2). Optimally, the tailoring and delivery of the appropriate therapy is carried out by a multidisciplinary team, including rheumatologists, nurses, psychologists, physiotherapists and occupational therapists, social workers, nutritionists, orthopaedic surgeons, and rehabilitation specialists.

The D2T concept encompasses all patients with RA whose symptom burden leads to diminished QoL, irrespective of underlying causes or inflammation status. Comprehensive assessment approaches are needed to understand the impact of disease on the patient’s life and to tailor interventions to individual needs and preferences. Alternative assessment methods, both subjective and objective, can be used to establish treatment targets for non-pharmacological therapies when composite disease activity indices (e.g. DAS28, CDAI) are less suitable [87]. The effectiveness of interventions can then be re-evaluated during follow-up visits based on the previously defined goals, guiding subsequent therapeutic decisions. The importance of individualized assessment and goal-setting is also reinforced by international guidelines [81, 83]. Various interventions address different dimensions of disease, e.g. pain (including nociplastic pain features), fatigue, mood disturbances [88], functional disability, or impaired work participation [87], which can be evaluated by PROs. For some PA interventions, performance-based tests and self-monitoring tools (e.g. wearables) may also be utilized [81].

**Figure 3:**
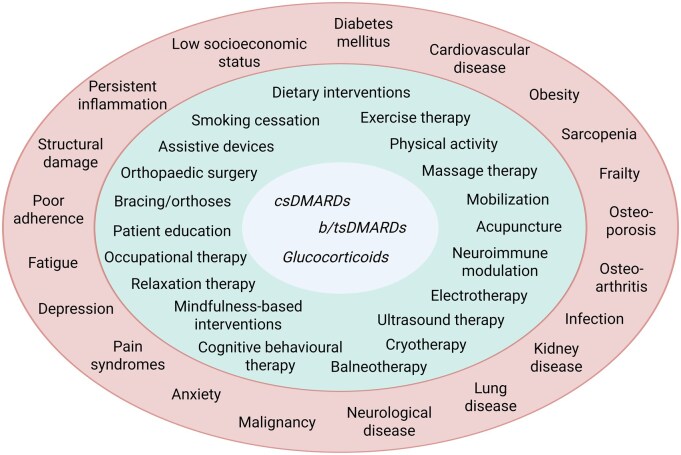
Examples of possible underlying factors of D2T RA (outer circle) and potential management options, including pharmacological and non-pharmacological interventions. A combination of factors (e.g. systemic inflammation, pain, fatigue, comorbidities, adherence problems) may drive pathogenesis and symptoms in the D2T state. Identifying these determinants can inform selection of the most suitable and safe pharmacological (inner circle) and non-pharmacological (middle circle) treatment options that comprise the individualized, multimodal ‘package of care’. b: biological; cs: conventional synthetic; D2T: difficult-to-treat; ts: targeted synthetic. Figure 3 was created in BioRender: Tóth, L. (2026) https://biorender.com/ioizxx1, accessed on 16 April 2026

In summary, recognizing the factors that perpetuate the D2T state can enhance the selection of specific treatment options (Fig. 3). We previously proposed a specific individualized strategy in D2T RA, combining standard rheumatology visits with routine assessments by a psychologist and a physiotherapist. Using this approach, specific interventions—e.g. educational programmes and psychotherapy, as well as exercise therapy—may be implemented, with their outcomes evaluated every 3–6 months [39].

General physical activity (PA) recommendations are applicable to patients with RA [81], and PA interventions, including exercise therapy, have repeatedly shown benefits for both RA-specific and systemic outcomes. Limited physical function and fatigue can play a role in sedentary behaviour, which is highly prevalent in RA and has been linked to increased disease activity [89]. Lifestyle interventions promoting physical activity are effective in increasing PA behaviour, as well as improving functional ability and fatigue [90, 91]. Several D2T contributors may be mitigated with PA interventions. Physical inactivity has been linked to an adverse CVD risk profile in RA, and PA may be effective in the improvement of several cardiovascular risk factors [92]. The benefits of regular exercise are highlighted in the EULAR recommendations for CVD risk management in RA [93], and a meta-analysis demonstrated the positive impact of aerobic exercise on cardiovascular fitness in RA and other rheumatic and musculoskeletal diseases (RMDs) [91]. Obesity is associated with worse outcomes in RA, and has been linked to the subsequent development of D2T disease [94]. In a trial of older people with RA and overweight/obesity, both a weight loss/exercise intervention and lifestyle counselling improved CVD risk profiles, with no significant difference between groups [95]. PA may also be valuable when RA is complicated with mental ill health. Meta-analyses have found that exercise was associated with reductions in anxiety as well as depressive symptoms in adults with RMDs (including RA and fibromyalgia) [96, 97]. Aerobic exercise programmes can also alleviate pain and fatigue and improve functional ability among patients with RA [98, 99].

Promoting and maintaining adherence to PA can be challenging over time; a previously proposed multidisciplinary team–led approach for the long-term sustainability of PA incorporates the establishment of individualized goals (e.g. step count) and their consistent reinforcement at subsequent visits [100]. Recommendations highlight the importance of identifying and addressing contraindications and barriers to engaging in PA [81], which are often more pronounced in D2T disease. For instance, sarcopenia, which is more prevalent in RA and contributes to physical disability, was recently associated with D2T status [101]. However, exercise therapy was found to increase muscle mass in patients with RA [102], suggesting that individualized interventions may still offer benefits. Other barriers include symptoms like pain, fatigue, stiffness, and kinesiophobia [103], which may be further intensified by comorbid conditions (osteoarthritis, pain syndromes, etc.).

Other modalities of physiotherapy—e.g. balneotherapy [104, 105], ultrasound therapy [106], or cryotherapy [107]—are sometimes employed in RA, although their efficacy and safety remains mostly unexplored due to a scarcity of clinical trials. Vagus nerve stimulation (VNS) is a novel approach that modulates the inflammatory reflex—a neural circuit regulating inflammatory responses—and early clinical studies suggested it was safe and may offer clinical improvements in patients with IR to multiple b/tsDMARDs [108, 109]. Recently, a pivotal randomized controlled trial (RCT) evaluated an implanted VNS system in 242 patients with IR to ≥1 targeted therapy, including 43% classified with D2T disease. The intervention achieved its primary efficacy end point—i.e. ACR20 response at 3 months—and showed further clinical benefits at 12 months along with a favourable safety profile [110].

Individuals with RA commonly experience mental ill health, and DMARDs tend to have a less pronounced impact on mental health compared with physical symptoms [111]. In a D2T RA cohort, the presence of mental health/pain–related illnesses adversely affected the long-term evolution of functional status and disease activity [112], underlining the importance of addressing psychosocial factors. Meta-analyses suggest that psychological interventions in RA may improve various outcomes [113]. Educational programmes have a positive impact on QoL in RA [114], and are widely recognized as an integral part of individualized care [82], providing patients with information on their condition. As mentioned in the EULAR PtC, providing education may support goal-setting for individuals with D2T disease when T2T recommendations are less feasible [31]. Some educational interventions have been found to enhance the self-management skills of patients starting or switching to a new b/tsDMARD [115, 116]. Education and combined self-management programmes contribute to self-efficacy [51], potentially improving mental health and treatment adherence in D2T patients. Cognitive behavioural therapy [117] and mindfulness-based interventions [118] have been associated with reductions in anxiety and depressive symptoms in RA, although their effect on pain levels remains less conclusive.

Some dietary interventions, such as specific diets or nutritional supplements, may have a positive effect on disease outcomes, including pain and disability [119, 120]. For instance, an RCT demonstrated that fish oil supplementation resulted in a reduced risk of triple csDMARD failure and a higher remission rate in patients with recent-onset RA [121]. However, evidence for the benefits of specific interventions remains limited, especially in the D2T population. The ACR guideline conditionally recommends adherence to the Mediterranean diet in RA based on its potential benefits on long-term health outcomes [85].

Recommendations underscore the importance of patient-tailored care provided by interprofessional teams to optimize treatment outcomes in RA [7, 85]. A long-standing intervention combining exercise therapy, education, and self-management was found to improve functional ability and quality of life among patients with RA and severe functional limitations [122], supporting the potential benefit of integrated care in D2T disease. Comprehensive rehabilitative approaches, including occupational therapy, may be applied to improve physical function and increase social participation [123]. Remarkably, telehealth approaches are increasingly applied in RA, and various interventions integrating digital tools have shown promising results in symptom control and self-management [124–126].

Despite these findings, data from routine clinical settings suggest that non-pharmacological approaches are often overlooked. In a study of nearly 200 000 RA patients from the USA, only 9.2% ever received a referral to physical therapy or occupational therapy from their rheumatology provider [127].

While the advent of effective pharmacotherapy has reduced surgical rates among RA patients [128], referral to an orthopaedic surgeon may support management of functional limitations in D2T disease [11, 39]. The Japan College of Rheumatology (JCR) developed a clinical practice guideline with a treatment algorithm and evidence-based recommendations corresponding to 19 clinical questions that concern surgical interventions and rehabilitation for patients with residual limb joint symptoms or functional impairment despite intensive drug treatment. The suggestions address the timing of surgery, indications of specific surgical methods, and surgery-related safety considerations [6, 84]. A Japanese propensity score–matched study evaluating the effect of orthopaedic surgical interventions in D2T and non-D2T RA patients indicated a postoperative improvement in disease activity, the patient’s assessment of general health, face scale, and Health Assessment Questionnaire—Disability Index (HAQ–DI) score [129]. Furthermore, the number of patients with improved HAQ–DI at 6 months and the magnitude of general health improvement at 6 and 12 months after intervention was higher in the D2T group [129].

## Future outlook

The challenges presented by D2T disease signal a need for more personalized approaches in RA care. The understanding of distinct pathogenic mechanisms underlying therapeutic failure at the patient level remains limited, and efforts to identify reliable markers for guiding therapeutic choices have so far proven unsuccessful. Given that a long-term perspective is essential for the treatment of RA, comparing the efficacy and safety of targeted therapies based on robust, longitudinal data will be important for optimizing treatment decisions. Innovative clinical trial designs can provide more direct insights into the links between treatment outcomes and molecular signatures [130]. For instance, in the biopsy-driven R4RA trial of patients with prior IR to TNF inhibitors, histological stratification as B cell–poor or B cell–rich did not reveal differences between rituximab and tocilizumab in either subtype; however, a reclassification via RNA sequencing showed that tocilizumab led to higher clinical response in those with low or absent B cell lineage expression signature [131]. Improved characterization of clinically relevant phenotypes—incorporating clinical traits, imaging and laboratory markers, and synovial signatures—may help establish mechanistically informed treatment algorithms and advance precision medicine [132].

The EULAR definition of D2T RA encompasses a wide patient population who have experienced multiple DMARD failures, without specifying the underlying causes. In recent years, various broader distinctions have been proposed within D2T disease—e.g. inflammatory vs non-inflammatory; IR vs adverse events—but their definitions remain inconsistent across studies. Future RCTs and prospective studies conducted in D2T populations should account for this heterogeneity and distinguish well-defined subgroups at baseline to improve insights into disease mechanisms and identify treatment response patterns associated with specific patient profiles [133].

In the future, new therapeutic choices may also facilitate therapeutic success in D2T disease. The combination of different targeted therapies to block multiple pathogenic pathways has been proposed as a promising strategy in D2T RA; however, the risk–benefit profile of such regimens remains ambiguous due to insufficient evidence, and they remain rarely used in clinical practice [134]. Furthermore, the immune reconstitution provided by chimeric antigen receptor (CAR) T cell therapy and bispecific T cell engagers has shown promise in autoimmune disease, suggesting possible application in D2T RA, although the efficacy and safety of these emerging therapeutics have yet to be established outside of case series [135, 136]. The evolution of mechanistic insights into RA pathogenesis continues to drive the exploration of novel treatment options; for example, synovial fibroblasts have recently emerged as attractive cellular targets for the therapy of refractory disease [137]. The recent success of VNS in a pivotal RCT (mentioned in the previous section) exemplifies the potential value of non-pharmacological approaches. To date, however, there is little formal evidence of the added benefits of non-drug therapy in D2T RA; therefore, their efficacy and safety should be further explored in trials and observational settings.

## Conclusion

In the management of RA, the T2T strategy has made remission a realistic treatment target by providing a structured algorithm for appropriate use of DMARDs and disease monitoring. However, the subgroup of patients who cycle through multiple targeted therapies without success still represent a substantial unmet medical need. The complexity of the D2T population requires an individualized approach, employing a combination of pharmacological and non-pharmacological interventions.

Some biological agents and JAK inhibitors maintain therapeutic benefits even in D2T RA patients and demonstrate efficacy independent of prior treatment. Real-world data from D2T populations indicate that JAK inhibitors may be particularly effective in controlling disease activity, and their concomitant analgesic actions reinforce their value in this patient group. However, high-quality studies (including RCTs) of targeted treatments specifically conducted in the D2T RA population are still largely lacking. Non-pharmacological interventions can be used as adjuncts at any stage of RA, especially if complaints of non-inflammatory nature are present, and they may have a particular value in settings where disease control cannot be achieved by available DMARDs. A more structured approach is needed for the delivery of non-pharmacological treatments in established RA, especially in D2T disease, with the adoption of alternative treatment targets.

In summary, managing D2T RA requires a comprehensive, multidisciplinary approach that combines carefully selected pharmacological strategies with structured non-pharmacological interventions. Well-designed, long-term studies are still needed to guide truly individualized care in this heterogeneous patient population.

## Data Availability

No new data were generated or analysed in support of this article.

## References

[1] Smolen JS , Aletaha D, Barton A et al Rheumatoid arthritis. Nat Rev Dis Primers 2018;4:18001. 29417936 10.1038/nrdp.2018.1

[2] Tanaka Y. Rheumatoid arthritis. Inflamm Regener 2020;40:20. 10.1186/s41232-020-00133-8 PMC7487964 32944095

[3] Finckh A , Gilbert B, Hodkinson B et al Global epidemiology of rheumatoid arthritis. Nat Rev Rheumatol 2022;18:591–602. 36068354 10.1038/s41584-022-00827-y

[4] Brown P , Pratt AG, Hyrich KL. Therapeutic advances in rheumatoid arthritis. BMJ. 2024;384:e070856. 38233032 10.1136/bmj-2022-070856

[5] Fraenkel L , Bathon JM, England BR et al 2021 American College of Rheumatology Guideline for the treatment of rheumatoid arthritis. Arthritis Rheumatol 2021;73:1108–23. 34101376 10.1002/art.41752

[6] Harigai M , Kaneko Y, Tanaka E et al 2024 update of the Japan College of Rheumatology Clinical Practice Guidelines for the Management of Rheumatoid Arthritis: secondary publication. Mod Rheumatol 2025;35:387–401. 39820350 10.1093/mr/roaf006

[7] Smolen JS , Edwards CJ, Konzett V et al EULAR recommendations for the management of rheumatoid arthritis with synthetic and biologic disease-modifying antirheumatic drugs: 2025 update. Ann Rheum Dis 2026. 10.1016/j.ard.2026.01.023 41826212

[8] Smolen JS , Breedveld FC, Burmester GR et al Treating rheumatoid arthritis to target: 2014 update of the recommendations of an international task force. Ann Rheum Dis 2016;75:3–15. 25969430 10.1136/annrheumdis-2015-207524 PMC4717393

[9] Stoffer MA , Schoels MM, Smolen JS et al Evidence for treating rheumatoid arthritis to target: results of a systematic literature search update. Ann Rheum Dis 2016;75:16–22. 25990290 10.1136/annrheumdis-2015-207526 PMC4717391

[10] Batko B , Batko K, Krzanowski M, Żuber Z. Physician adherence to treat-to-target and practice guidelines in rheumatoid arthritis. J Clin Med 2019;8:1416. 31500394 10.3390/jcm8091416 PMC6780913

[11] de Hair MJH , Jacobs JWG, Schoneveld JLM, van Laar JM. Difficult-to-treat rheumatoid arthritis: an area of unmet clinical need. Rheumatology 2017;57:1135–44. 10.1093/rheumatology/kex349 29029308

[12] Nagy G , Roodenrijs NM, Welsing PM et al EULAR definition of difficult-to-treat rheumatoid arthritis. Ann Rheum Dis 2021;80:31–5. 33004335 10.1136/annrheumdis-2020-217344 PMC7788062

[13] Xie W , Chen T, Xiao S, Huang H, Zhang Z. Difficult-to-treat rheumatoid arthritis: systematic review and meta-analysis on global prevalence. Ann Rheum Dis 2026;85:417–24. 41188120 10.1016/j.ard.2025.10.007

[14] Nagy G , Gunkl-Tóth L, Dorgó AM, McInnes IB. The concept of difficult-to-treat disease in rheumatology: where next? Lancet Rheumatol 2025;7:e274–e89. 39848270 10.1016/S2665-9913(24)00340-0

[15] Dorgó AM , Gunkl-Tóth L, Nagy G. Pathogenic drivers of difficult-to-treat rheumatoid arthritis: synovium and beyond. Int J Mol Sci 2026;27:1860. 41751996 10.3390/ijms27041860 PMC12940380

[16] Dey M , Nagy G, Nikiphorou E. Comorbidities and extra-articular manifestations in difficult-to-treat rheumatoid arthritis: different sides of the same coin? Rheumatology 2023;62:1773–9. 36205537 10.1093/rheumatology/keac584

[17] Hofman ZLM , Roodenrijs NMT, Nikiphorou E et al Difficult-to-treat rheumatoid arthritis: what have we learned and what do we still need to learn? Rheumatology 2025;64:65–73. 39383505 10.1093/rheumatology/keae544 PMC11701314

[18] Novella-Navarro M , Balsa A. Difficult-to-treat rheumatoid arthritis in older adults: implications of ageing for managing patients. Drugs Aging 2022;39:841–9. 36104655 10.1007/s40266-022-00976-5 PMC9626415

[19] Lehoczki A , Ungvari Z, Szappanos Á et al Geroscience insights into difficult-to-treat rheumatoid arthritis: the role of unhealthy aging, comorbidity, and therapeutic complexity. GeroScience 2026;48:2153–77. 41454175 10.1007/s11357-025-02049-y PMC12972182

[20] Takanashi S , Kaneko Y, Takeuchi T. Elderly patients with comorbidities in the definition of difficult-to-treat rheumatoid arthritis. Ann Rheum Dis 2021;80:1491–3. 33962961 10.1136/annrheumdis-2021-220315

[21] Jung J-Y , Lee E, Kim J-W et al Difficult-to-treat rheumatoid arthritis among elderly patients from the KOBIO registry. Eur J Intern Med 2025;137:96–104. 40328520 10.1016/j.ejim.2025.04.040

[22] Roodenrijs NMT , van der Goes MC, Welsing PMJ et al Non-adherence in difficult-to-treat rheumatoid arthritis from the perspectives of patients and rheumatologists: a concept mapping study. Rheumatology 2021;60:5105–16. 33560301 10.1093/rheumatology/keab130

[23] Molina E , del Rincon I, Restrepo JF, Battafarano DF, Escalante A. Association of socioeconomic status with treatment delays, disease activity, joint damage, and disability in rheumatoid arthritis. Arthritis Care Res 2015;67:940–6. 10.1002/acr.22542 PMC4482767 25581770

[24] Boytsov N , Zhang X, Evans KA, Johnson BH. Impact of plan-level access restrictions on effectiveness of biologics among patients with rheumatoid or psoriatic arthritis. PharmacoEconomics – Open 2020;4:105–17. 31177506 10.1007/s41669-019-0152-1 PMC7018889

[25] Takanashi S , Kaneko Y, Takeuchi T. Characteristics of patients with difficult-to-treat rheumatoid arthritis in clinical practice. Rheumatology 2021;60:5247–56. 33682890 10.1093/rheumatology/keab209

[26] Giollo A , Zen M, Larosa M et al Early characterization of difficult-to-treat rheumatoid arthritis by suboptimal initial management: a multicentre cohort study. Rheumatology 2023;62:2083–9. 36190344 10.1093/rheumatology/keac563

[27] Roodenrijs NMT , van der Goes MC, Welsing PMJ et al Difficult-to-treat rheumatoid arthritis: contributing factors and burden of disease. Rheumatology 2021;60:3778–88. 33331946 10.1093/rheumatology/keaa860

[28] Buch MH , Eyre S, McGonagle D. Persistent inflammatory and non-inflammatory mechanisms in refractory rheumatoid arthritis. Nat Rev Rheumatol 2021;17:17–33. 33293696 10.1038/s41584-020-00541-7

[29] Takanashi S , Takeuchi T, Kaneko Y. Five-year follow-up of patients with difficult-to-treat rheumatoid arthritis. Rheumatology 2025;64:2487–95. 38851883 10.1093/rheumatology/keae325 PMC12048047

[30] Cincinelli G , Maioli G, Posio C et al Truth unveiled by time and the marbled definition of D2T-RA: retrospective analysis on the persistence of the difficult-to-treat status among refractory RA patients. Arthritis Res Ther 2024;26:161. 39289770 10.1186/s13075-024-03390-x PMC11406730

[31] Nagy G , Roodenrijs NMT, Welsing PMJ et al EULAR points to consider for the management of difficult-to-treat rheumatoid arthritis. Ann Rheum Dis 2022;81:20–33. 34407926 10.1136/annrheumdis-2021-220973 PMC8761998

[32] Roodenrijs NMT , Kedves M, Hamar A et al Diagnostic issues in difficult-to-treat rheumatoid arthritis: a systematic literature review informing the EULAR recommendations for the management of difficult-to-treat rheumatoid arthritis. RMD Open 2021;7:e001511. 33514671 10.1136/rmdopen-2020-001511 PMC7849901

[33] Bellis E , Agugliaro F, Garulli C et al The role of musculoskeletal ultrasound in difficult-to-treat RA: insights from a systematic literature review. Autoimmun Rev 2025;24:103694. 39557317 10.1016/j.autrev.2024.103694

[34] Garcia-Salinas R , Sanchez-Prado E, Mareco J et al Difficult to treat rheumatoid arthritis in a comprehensive evaluation program: frequency according to different objective evaluations. Rheumatol Int 2023;43:1821–8. 37269430 10.1007/s00296-023-05349-8

[35] David P , Di Matteo A, Hen O et al Poly-refractory rheumatoid arthritis: an uncommon subset of difficult to treat disease with distinct inflammatory and noninflammatory phenotypes. Arthritis Rheumatol 2024;76:510–21. 38059326 10.1002/art.42767

[36] Tan Y , Rogers G, Shukla R, Ho P, Buch MH. Characterising subgroups of difficult-to-treat rheumatoid arthritis in real-world clinical settings. EULAR Rheumatol Open 2026;2:36–41. 10.1016/j.ero.2025.11.024 PMC13292293 42370095

[37] Giollo A , Salvato M, Frizzera F et al Clinical application of synovial biopsy in noninflammatory and persistent inflammatory refractory rheumatoid arthritis. Ann Rheum Dis 2026;85:91–102. 40846588 10.1016/j.ard.2025.07.023

[38] Sakai H , Sonomoto K, Nakayamada S et al Real-world effectiveness of b/tsDMARD switching in low-inflammatory difficult-to-treat rheumatoid arthritis: insights from the FIRST registry. RMD Open 2026;12:e006513. 41644272 10.1136/rmdopen-2025-006513 PMC12878399

[39] Majnik J , Császár-Nagy N, Böcskei G, Bender T, Nagy G. Non-pharmacological treatment in difficult-to-treat rheumatoid arthritis. Front Med 2022;9:991677. 10.3389/fmed.2022.991677 PMC9465607 36106320

[40] Emery P , Keystone E, Tony HP et al IL-6 receptor inhibition with tocilizumab improves treatment outcomes in patients with rheumatoid arthritis refractory to anti-tumour necrosis factor biologicals: results from a 24-week multicentre randomised placebo-controlled trial. Ann Rheum Dis 2008;67:1516–23. 18625622 10.1136/ard.2008.092932 PMC3811149

[41] Rubbert-Roth A , Enejosa J, Pangan AL et al Trial of upadacitinib or abatacept in rheumatoid arthritis. N Engl J Med 2020;383:1511–21. 33053283 10.1056/NEJMoa2008250

[42] Novella-Navarro M , Ruiz-Esquide V, López-Juanes N et al Subsequent biologic and targeted synthetic disease modifying anti rheumatic drugs after fulfilling difficult-to-treat rheumatoid arthritis criteria: a survival analysis. Clin Rheumatol 2024;43:2817–23. 39009920 10.1007/s10067-024-07070-8

[43] Hecquet S , Combier A, Steelandt A et al Characteristics of patients with difficult-to-treat rheumatoid arthritis in a French single-centre hospital. Rheumatology 2023;62:3866–74. 36961324 10.1093/rheumatology/kead143

[44] Genovese MC , Fleischmann R, Combe B et al Safety and efficacy of upadacitinib in patients with active rheumatoid arthritis refractory to biologic disease-modifying anti-rheumatic drugs (SELECT-BEYOND): a double-blind, randomised controlled phase 3 trial. Lancet 2018;391:2513–24. 29908670 10.1016/S0140-6736(18)31116-4

[45] Genovese MC , Kalunian K, Gottenberg J-E et al Effect of filgotinib vs placebo on clinical response in patients with moderate to severe rheumatoid arthritis refractory to disease-modifying antirheumatic drug therapy: the FINCH 2 randomized clinical trial. JAMA 2019;322:315–25. 31334793 10.1001/jama.2019.9055 PMC6652745

[46] Genovese MC , Kremer JM, Kartman CE et al Response to baricitinib based on prior biologic use in patients with refractory rheumatoid arthritis. Rheumatology 2018;57:900–8. 29415145 10.1093/rheumatology/kex489 PMC5913638

[47] Al Tabaa O , Hecquet S, Thomas M et al Real-world assessment of the efficacy and tolerability profile of JAK inhibitors in difficult-to-treat rheumatoid arthritis. Sem Arthritis Rheum 2024;69:152572. 10.1016/j.semarthrit.2024.152572 39509839

[48] Hayashi S , Nakano N, Tsubosaka M et al Real-world study comparing the efficacy of Janus kinase inhibitors in patients with difficult-to-treat rheumatoid arthritis. Clin Rheumatol 2024;43:3285–92. 39243280 10.1007/s10067-024-07117-w

[49] Ochi S , Mizoguchi F, Nakano K, Tanaka Y. Difficult-to-treat rheumatoid arthritis with respect to responsiveness to biologic/targeted synthetic DMARDs: a retrospective cohort study from the FIRST registry. Clin Exp Rheumatol 2022;40:86–96. 10.55563/clinexprheumatol/g33ia5 33635223

[50] Zhao SS , Kearsley-Fleet L, Bosworth A, BSRBR-RA Contributors Group et al Effectiveness of sequential biologic and targeted disease modifying anti-rheumatic drugs for rheumatoid arthritis. Rheumatology 2022;61:4678–86. 35357421 10.1093/rheumatology/keac190 PMC9707051

[51] Roodenrijs NMT , Hamar A, Kedves M et al Pharmacological and non-pharmacological therapeutic strategies in difficult-to-treat rheumatoid arthritis: a systematic literature review informing the EULAR recommendations for the management of difficult-to-treat rheumatoid arthritis. RMD Open 2021;7:e001512. 33419871 10.1136/rmdopen-2020-001512 PMC7798678

[52] Yoshii I , Sawada N, Chijiwa T. Clinical characteristics and variants that predict prognosis of difficult-to-treat rheumatoid arthritis. Rheumatol Int 2022;42:1947–54. 35410410 10.1007/s00296-022-05124-1

[53] Jung J-Y , Lee E, Kim J-W et al Unveiling difficult-to-treat rheumatoid arthritis: long-term impact of biologic or targeted synthetic DMARDs from the KOBIO registry. Arthritis Res Ther 2023;25:174. 37726808 10.1186/s13075-023-03165-w PMC10507947

[54] Ochi S , Sonomoto K, Nakayamada S, Tanaka Y. Preferable outcome of Janus kinase inhibitors for a group of difficult-to-treat rheumatoid arthritis patients: from the FIRST Registry. Arthritis Res Ther 2022;24:61. 35232462 10.1186/s13075-022-02744-7 PMC8886884

[55] Migliore A , Pompilio G, Integlia D, Zhuo J, Alemao E. Cycling of tumor necrosis factor inhibitors versus switching to different mechanism of action therapy in rheumatoid arthritis patients with inadequate response to tumor necrosis factor inhibitors: a Bayesian network meta-analysis. Ther Adv Musculoskelet Dis 2021;13:1759720X211002682. 10.1177/1759720X211002682 PMC8010806 33854570

[56] Pombo-Suarez M , Sanchez-Piedra C, Gómez-Reino J et al After JAK inhibitor failure: to cycle or to switch, that is the question–data from the JAK-pot collaboration of registries. Ann Rheum Dis 2023;82:175–81. 36100351 10.1136/ard-2022-222835

[57] Watanabe R , Ebina K, Gon T et al Predictive factors and treatment outcomes associated with difficult-to-treat rheumatoid arthritis conditions: the ANSWER cohort study. Rheumatology 2024;63:2418–26. 38724245 10.1093/rheumatology/keae265

[58] Tóth L , Juhász MF, Szabó L et al Janus kinase inhibitors improve disease activity and patient-reported outcomes in rheumatoid arthritis: a systematic review and meta-analysis of 24,135 patients. Int J Mol Sci 2022;23:1246. 35163173 10.3390/ijms23031246 PMC8836107

[59] Eberhard A , Di Giuseppe D, Askling J et al Effectiveness of JAK inhibitors compared with biologic disease-modifying antirheumatic drugs on pain reduction in rheumatoid arthritis: results from a nationwide Swedish Cohort Study. Arthritis Rheumatol 2025;77:253–62. 39308007 10.1002/art.43014 PMC11865685

[60] Xiong Y , Song X, Sheng X et al A review of Janus kinase/signal transducer and activator of transcription signaling and cytokines in the pain mechanism of rheumatoid arthritis. Eur J Inflamm 2023;21:1721727X231197498.

[61] Hu X , li J, Fu M, Zhao X, Wang W. The JAK/STAT signaling pathway: from bench to clinic. Signal Transduct Target Ther 2021;6:402. 34824210 10.1038/s41392-021-00791-1 PMC8617206

[62] Stefanov K , McGucken A, Arnott M et al JAK inhibition appears to alter clinical and neurobiological markers of nociplastic pain in rheumatoid arthritis: a 7T MRI Brain study [abstract]. Arthritis Rheumatol 2025;77. https://acrabstracts.org/abstract/jak-inhibition-appears-to-alter-clinical-and-neurobiological-markers-of-nociplastic-pain-in-rheumatoid-arthritis-a-7t-mri-brain-study/

[63] Basu N , Al-Wasity S, Brock J et al POS0310: baricitinib may reduce co-existing fibromyalgia in people with rheumatoid arthritis: an ultra-high resolution MRI brain study [abstract]. Ann Rheum Dis 2024;83:320.

[64] Hess A , Axmann R, Rech J et al Blockade of TNF-α rapidly inhibits pain responses in the central nervous system. Proc Natl Acad Sci USA 2011;108:3731–6. 21245297 10.1073/pnas.1011774108 PMC3048151

[65] Trouvin A-P , Simunek A, Coste J et al Changes in descending pain modulation during anti–tumor necrosis factor therapy: a prospective study in rheumatoid arthritis and spondyloarthritis. Arthritis Rheumatol 2025;77:658–63. 39679776 10.1002/art.43084 PMC12123248

[66] Rech J , Hess A, Finzel S et al Association of brain functional magnetic resonance activity with response to tumor necrosis factor inhibition in rheumatoid arthritis. Arthritis Rheum 2013;65:325–33. 23238986 10.1002/art.37761

[67] Paudel ML , Li R, Naik C et al Prevalence and characteristics of adults with difficult-to-treat rheumatoid arthritis in a large patient registry. Rheumatology 2025;64:1102–10. 38837701 10.1093/rheumatology/keae318 PMC11879286

[68] Singh JA , Cameron C, Noorbaloochi S et al Risk of serious infection in biological treatment of patients with rheumatoid arthritis: a systematic review and meta-analysis. Lancet 2015;386:258–65. 25975452 10.1016/S0140-6736(14)61704-9 PMC4580232

[69] Konzett V , Aletaha D. Management strategies in rheumatoid arthritis. Nat Rev Rheumatol 2024;20:760–9. 39448800 10.1038/s41584-024-01169-7

[70] Nash P , Kerschbaumer A, Konzett V et al Expert consensus statement on the treatment of immune-mediated inflammatory diseases with Janus kinase inhibitors: 2024 update. Ann Rheum Dis 2025;84:664–79. 40037995 10.1016/j.ard.2025.01.032

[71] Holroyd CR , Seth R, Bukhari M et al The British Society for Rheumatology biologic DMARD safety guidelines in inflammatory arthritis. Rheumatology 2019;58:e3–e42. 30137552 10.1093/rheumatology/key208

[72] Sebbag E , Lauper K, Molina-Collada J et al EULAR points to consider on the initiation of targeted therapies in patients with inflammatory arthritis and a history of cancer. Ann Rheum Dis 2025;84:388–97. 10.1136/ard-2024-225982 39739385

[73] Ytterberg SR , Bhatt DL, Mikuls TR et al; ORAL Surveillance Investigators. Cardiovascular and cancer risk with tofacitinib in rheumatoid arthritis. N Engl J Med 2022;386:316–26. 35081280 10.1056/NEJMoa2109927

[74] Curtis JR , Yamaoka K, Chen Y-H et al Malignancy risk with tofacitinib versus TNF inhibitors in rheumatoid arthritis: results from the open-label, randomised controlled ORAL Surveillance trial. Ann Rheum Dis 2023;82:331–43. 36600185 10.1136/ard-2022-222543 PMC9933177

[75] Szekanecz Z , Buch MH, Charles-Schoeman C et al Efficacy and safety of JAK inhibitors in rheumatoid arthritis: update for the practising clinician. Nat Rev Rheumatol 2024;20:101–15. 38216757 10.1038/s41584-023-01062-9

[76] Sonomoto K , Tanaka Y. Malignancies and rheumatoid arthritis, csDMARDs, biological DMARDs, and JAK inhibitors: challenge and outlook. Exp Rev Clin Immunol 2023;19:1325–42. 10.1080/1744666X.2023.2247158 37578325

[77] Miyata H , Sonomoto K, Fukuyo S et al Computed tomography for malignancy screening in patients with rheumatoid arthritis before initiation of disease modifying antirheumatic drug. Rheumatology 2023;62:3339–49. 36782362 10.1093/rheumatology/kead075

[78] Funada M , Miyazaki Y, Nakayamada S et al CT informs detection and treatment options in rheumatoid arthritis complicated by pulmonary non-tuberculous mycobacterial disease from the FIRST registry. RMD Open 2024;10:e004049. 38866590 10.1136/rmdopen-2023-004049 PMC11177696

[79] Sonomoto K , Tanaka Y. Targeted therapies for rheumatoid arthritis in super-elderly society: insights from FIRST Registry, Japan. Int J Rheum Dis 2025;28:e70232. 40275579 10.1111/1756-185X.70232 PMC12022466

[80] Sonomoto K , Nakayamada S, Tanaka H, Nagayasu A, Tanaka Y. Real-world safety and efficacy of targeted therapies in rheumatoid arthritis: a 5-year, 5130-case follow-up from FIRST registry. Rheumatol Ther 2025;12:561–80. 40257743 10.1007/s40744-025-00762-w PMC12084202

[81] Rausch Osthoff A-K , Niedermann K, Braun J et al 2018 EULAR recommendations for physical activity in people with inflammatory arthritis and osteoarthritis. Ann Rheum Dis 2018;77:1251–60. 29997112 10.1136/annrheumdis-2018-213585

[82] Zangi HA , Ndosi M, Adams J et al; European League Against Rheumatism (EULAR). EULAR recommendations for patient education for people with inflammatory arthritis. Ann Rheum Dis 2015;74:954–62. 25735643 10.1136/annrheumdis-2014-206807

[83] Nikiphorou E , Santos EJF, Marques A et al 2021 EULAR recommendations for the implementation of self-management strategies in patients with inflammatory arthritis. Ann Rheum Dis 2021;80:1278–85. 33962964 10.1136/annrheumdis-2021-220249 PMC8458093

[84] Ito H , Nishida K, Kojima T et al Non-drug and surgical treatment algorithm and recommendations for the 2020 update of the Japan College of Rheumatology Clinical Practice Guidelines for the Management of Rheumatoid Arthritis—secondary publication. Mod Rheumatol 2023;33:36–45. 35294030 10.1093/mr/roac019

[85] England BR , Smith BJ, Baker NA et al 2022 American College of Rheumatology Guideline for Exercise, Rehabilitation, Diet, and Additional Integrative Interventions for Rheumatoid Arthritis. Arthritis Care Res 2023;75:1603–15. 10.1002/acr.25117 37227116

[86] Smolen JS , Aletaha D, Bijlsma JWJ et al; T2T Expert Committee. Treating rheumatoid arthritis to target: recommendations of an international task force. Ann Rheum Dis 2010;69:631–7. 20215140 10.1136/ard.2009.123919 PMC3015099

[87] Küçükdeveci AA. Nonpharmacological treatment in established rheumatoid arthritis. Best Pract Res Clin Rheumatol 2019;33:101482. 31987686 10.1016/j.berh.2019.101482

[88] Kelleher EM , Meouchi R, Irani A. Beyond inflammation: why understanding the brain matters in inflammatory arthritis. Arthritis Care Res 2026;78:3–14. 10.1002/acr.25694 PMC12826092 41236146

[89] Fenton SAM , Veldhuijzen van Zanten JJCS, Duda JL, Metsios GS, Kitas GD. Sedentary behaviour in rheumatoid arthritis: definition, measurement and implications for health. Rheumatology 2018;57:213–26. 28398519 10.1093/rheumatology/kex053

[90] Brady SM , Veldhuijzen van Zanten JJCS, Dinas PC et al Effects of lifestyle physical activity and sedentary behaviour interventions on disease activity and patient- and clinician-important health outcomes in rheumatoid arthritis: a systematic review with meta-analysis. BMC Rheumatol 2023;7:27. 37674187 10.1186/s41927-023-00352-9 PMC10481589

[91] Rausch Osthoff A-K , Juhl CB, Knittle K et al Effects of exercise and physical activity promotion: meta-analysis informing the 2018 EULAR recommendations for physical activity in people with rheumatoid arthritis, spondyloarthritis and hip/knee osteoarthritis. RMD Open 2018;4:e000713. 30622734 10.1136/rmdopen-2018-000713 PMC6307596

[92] Metsios GS , Moe RH, van der Esch M et al; IMPACT-RMD Consortium. The effects of exercise on cardiovascular disease risk factors and cardiovascular physiology in rheumatoid arthritis. Rheumatol Int 2020;40:347–57. 31802210 10.1007/s00296-019-04483-6

[93] Agca R , Heslinga SC, Rollefstad S et al EULAR recommendations for cardiovascular disease risk management in patients with rheumatoid arthritis and other forms of inflammatory joint disorders: 2015/2016 update. Ann Rheum Dis 2017;76:17–28. 27697765 10.1136/annrheumdis-2016-209775

[94] Luciano N , Barone E, Brunetta E et al Obesity and fibromyalgia are associated with difficult-to-treat rheumatoid arthritis (D2T-RA) independent of age and gender. Arthritis Res Ther 2025;27:2. 39754234 10.1186/s13075-024-03432-4 PMC11697877

[95] Andonian BJ , Ross LM, Sudnick AM et al Effect of remotely supervised weight loss and exercise training versus lifestyle counseling on cardiovascular risk and clinical outcomes in older adults with rheumatoid arthritis: a randomized controlled trial. ACR Open Rheumatol 2024;6:124–36. 38126260 10.1002/acr2.11639 PMC10933621

[96] Kelley GA , Kelley KS, Callahan LF. Community-deliverable exercise and anxiety in adults with arthritis and other rheumatic diseases: a systematic review with meta-analysis of randomised controlled trials. BMJ Open 2018;8:e019138. 10.1136/bmjopen-2017-019138 PMC5855450 29455165

[97] Kelley GA , Kelley KS, Hootman JM. Effects of exercise on depression in adults with arthritis: a systematic review with meta-analysis of randomized controlled trials. Arthritis Res Ther 2015;17:21. 25645739 10.1186/s13075-015-0533-5 PMC4467075

[98] Ye H , Weng H, Xu Y et al Effectiveness and safety of aerobic exercise for rheumatoid arthritis: a systematic review and meta-analysis of randomized controlled trials. BMC Sports Sci Med Rehabil 2022;14:17. 35123568 10.1186/s13102-022-00408-2 PMC8818158

[99] Rongen-van Dartel SAA , Repping-Wuts H, Flendrie M et al Effect of aerobic exercise training on fatigue in rheumatoid arthritis: a meta-analysis. Arthritis Care Res 2015;67:1054–62. 10.1002/acr.22561 25624016

[100] Metsios GS , Kitas GD. Physical activity, exercise and rheumatoid arthritis: effectiveness, mechanisms and implementation. Best Pract Res Clin Rheumatol 2018;32:669–82. 31203925 10.1016/j.berh.2019.03.013

[101] Ohashi Y , Suzuki M, Sobue Y et al The association between difficult-to-treat rheumatoid arthritis and probable sarcopenia. Mod Rheumatol 2025;35:410–6. 39780522 10.1093/mr/roae116

[102] Liao C-D , Chen H-C, Huang S-W, Liou T-H. Exercise therapy for sarcopenia in rheumatoid arthritis: a meta-analysis and meta-regression of randomized controlled trials. Clin Rehabil 2022;36:145–57. 34404254 10.1177/02692155211035539

[103] Veldhuijzen van Zanten JJCS , Rouse PC, Hale ED et al Perceived barriers, facilitators and benefits for regular physical activity and exercise in patients with rheumatoid arthritis: a review of the literature. Sports Med 2015;45:1401–12. 26219268 10.1007/s40279-015-0363-2 PMC4579262

[104] Fernandez-Gonzalez M , Fernandez-Lao C, Martin-Martin L et al Therapeutic benefits of balneotherapy on quality of life of patients with rheumatoid arthritis: a systematic review. Int J Environ Res Public Health 2021;18:13216. 34948827 10.3390/ijerph182413216 PMC8701266

[105] Verhagen AP , Bierma-Zeinstra SMA, Boers M et al; Cochrane Musculoskeletal Group. Balneotherapy (or spa therapy) for rheumatoid arthritis. Cochrane Database Syst Rev 2015;2015:CD000518. 10.1002/14651858.CD000518.pub2 PMC7045434 25862243

[106] Király M , Varga Z, Szanyó F et al Effects of underwater ultrasound therapy on pain, inflammation, hand function and quality of life in patients with rheumatoid arthritis–a randomized controlled trial. Braz J Phys Ther 2017;21:199–205. 28442212 10.1016/j.bjpt.2017.04.002 PMC5537462

[107] Yao Y , Xie W, Opoku M et al Cryotherapy and thermotherapy in the management of osteoarthritis and rheumatoid arthritis: a comprehensive review. Fundam Res 2025;5:2409–31. 41466979 10.1016/j.fmre.2024.07.008 PMC12744633

[108] Koopman FA , Chavan SS, Miljko S et al Vagus nerve stimulation inhibits cytokine production and attenuates disease severity in rheumatoid arthritis. Proc Natl Acad Sci 2016;113:8284–9. 27382171 10.1073/pnas.1605635113 PMC4961187

[109] Genovese MC , Gaylis NB, Sikes D et al Safety and efficacy of neurostimulation with a miniaturised vagus nerve stimulation device in patients with multidrug-refractory rheumatoid arthritis: a two-stage multicentre, randomised pilot study. Lancet Rheumatol 2020;2:e527–e38. 38273617 10.1016/S2665-9913(20)30172-7

[110] Tesser JRP , Crowley AR, Box EJ et al Vagus nerve-mediated neuroimmune modulation for rheumatoid arthritis: a pivotal randomized controlled trial. Nat Med 2026;32:369–78. 41429981 10.1038/s41591-025-04114-7 PMC12823386

[111] Matcham F , Galloway J, Hotopf M et al The impact of targeted rheumatoid arthritis pharmacologic treatment on mental health. Arthritis Rheumatol 2018;70:1377–91. 29873196 10.1002/art.40565

[112] Antonios B , Irini DF, Argyro R et al Patterns of comorbidities differentially affect long-term functional evolution and disease activity in patients with 'difficult to treat’ rheumatoid arthritis. RMD Open 2024;10:e003808. 38242549 10.1136/rmdopen-2023-003808 PMC10806522

[113] Nagy Z , Szigedi E, Takács S, Császár-Nagy N. The effectiveness of psychological interventions for rheumatoid arthritis (RA): a systematic review and meta-analysis. Life 2023;13:849. 36984004 10.3390/life13030849 PMC10057722

[114] Bounabe A , Elammare S, Janani S, Ouabich R, Elarrachi I. Effectiveness of patient education on the quality of life of patients with rheumatoid arthritis: a systematic review and meta-analysis. Semin Arthritis Rheum 2024;69:152569. 39423700 10.1016/j.semarthrit.2024.152569

[115] Li LC , Shaw CD, Lacaille D et al Effects of a web-based patient decision aid on biologic and small-molecule agents for rheumatoid arthritis: results from a proof-of-concept study. Arthritis Care Res 2018;70:343–52. 10.1002/acr.23287 28544648

[116] Beauvais C , Fayet F, Rousseau A et al Efficacy of a nurse-led patient education intervention in promoting safety skills of patients with inflammatory arthritis treated with biologics: a multicentre randomised clinical trial. RMD Open 2022;8:e001828. 35296528 10.1136/rmdopen-2021-001828 PMC8928395

[117] Shen B , Li Y, Du X et al Effects of cognitive behavioral therapy for patients with rheumatoid arthritis: a systematic review and meta-analysis. Psychol Health Med 2020;25:1179–91. 32129673 10.1080/13548506.2020.1736312

[118] DiRenzo D , Crespo-Bosque M, Gould N et al Systematic review and meta-analysis: mindfulness-based interventions for rheumatoid arthritis. Curr Rheumatol Rep 2018;20:75. 30338418 10.1007/s11926-018-0787-4 PMC6233984

[119] Philippou E , Petersson SD, Rodomar C, Nikiphorou E. Rheumatoid arthritis and dietary interventions: systematic review of clinical trials. Nutr Rev 2021;79:410–28. 32585000 10.1093/nutrit/nuaa033

[120] Turk MA , Liu Y, Pope JE. Non-pharmacological interventions in the treatment of rheumatoid arthritis: a systematic review and meta-analysis. Autoimmun Rev 2023;22:103323. 36940841 10.1016/j.autrev.2023.103323

[121] Proudman SM , James MJ, Spargo LD et al Fish oil in recent onset rheumatoid arthritis: a randomised, double-blind controlled trial within algorithm-based drug use. Ann Rheum Dis 2015;74:89–95. 24081439 10.1136/annrheumdis-2013-204145

[122] Teuwen MMH , van Weely SFE, Vliet Vlieland TPM et al Effectiveness of longstanding exercise therapy compared with usual care for people with rheumatoid arthritis and severe functional limitations: a randomised controlled trial. Ann Rheum Dis 2024;83:437–45. 38171602 10.1136/ard-2023-224912

[123] Allameen NA , Lai YW, Lian G et al Physiotherapy and occupational therapy in rheumatoid arthritis: bridging functional and comorbidity gaps. Best Pract Res Clin Rheumatol 2025;39:102032. 39743473 10.1016/j.berh.2024.102032

[124] Betz LT , Jacob GA, Knitza J, Koehm M, Behrens F. Efficacy of a cognitive-behavioral digital therapeutic on psychosocial outcomes in rheumatoid arthritis: randomized controlled trial. NPJ Ment Health Res 2024;3:41. 39227501 10.1038/s44184-024-00085-8 PMC11371912

[125] Li C , Huang J, Wu H et al Management of rheumatoid arthritis with a digital health application: a multicenter, pragmatic randomized clinical trial. JAMA Netw Open 2023;6:e238343–e. 37058302 10.1001/jamanetworkopen.2023.8343 PMC10105314

[126] Li LC , Xie H, Feehan LM et al Effect of digital monitoring and counselling on self-management ability in patients with rheumatoid arthritis: a randomised controlled trial. Rheumatology 2025;64:310–20. 38152927 10.1093/rheumatology/kead709 PMC11701315

[127] Thoma L , Li J, Schmajuk G. Examination of rehabilitation referrals from rheumatology practices for adults with rheumatoid arthritis in the rise registry: a feasibility and descriptive study [abstract]. Arthritis Rheumatol 2024;76:3970–2.

[128] Nikiphorou E , Konan S, MacGregor AJ, Haddad FS, Young A. The surgical treatment of rheumatoid arthritis. Bone Joint J 2014;96-B:1287–9. 25274910 10.1302/0301-620X.96B10.34506

[129] Toyama S , Ishikawa H, Abe A et al Impact of orthopaedic surgical intervention on difficult-to-treat rheumatoid arthritis: a propensity score-matched study. Mod Rheumatol 2025;35:434–42. 39470370 10.1093/mr/roae097

[130] Pitzalis C , Choy EHS, Buch MH. Transforming clinical trials in rheumatology: towards patient-centric precision medicine. Nat Rev Rheumatol 2020;16:590–9. 32887976 10.1038/s41584-020-0491-4

[131] Humby F , Durez P, Buch MH et al; R4RA collaborative group. Rituximab versus tocilizumab in anti-TNF inadequate responder patients with rheumatoid arthritis (R4RA): 16-week outcomes of a stratified, biopsy-driven, multicentre, open-label, phase 4 randomised controlled trial. Lancet 2021;397:305–17. 33485455 10.1016/S0140-6736(20)32341-2 PMC7829614

[132] Gunkl-Tóth L , McInnes IB, Nagy G. Bridging the gap: combining treat-to-target and difficult-to-treat strategies in the management of rheumatoid arthritis. Nat Rev Rheumatol 2026;22:319–27. 41772290 10.1038/s41584-026-01354-w

[133] Nagy G , Buch MH. Strengths and limitations of the EULAR definition for difficult-to-treat rheumatoid arthritis. RMD Open 2025;11:e006271. 41224490 10.1136/rmdopen-2025-006271 PMC12612726

[134] Lignou AZ , Vassilakis KD, Baraliakos X et al Combination targeted therapy with two biologic/targeted synthetic DMARDs in 1200 patients with immune mediated inflammatory diseases. A systematic literature review for current landscape in safety and efficacy. Autoimmun Rev 2025;24:103865. 40633795 10.1016/j.autrev.2025.103865

[135] Freeley M. CAR T cell therapy for rheumatoid arthritis. Clin Rev Allergy Immunol 2025;68:100. 41258618 10.1007/s12016-025-09113-7 PMC12630179

[136] Bucci L , Hagen M, Rothe T et al Bispecific T cell engager therapy for refractory rheumatoid arthritis. Nat Med 2024;30:1593–601. 38671240 10.1038/s41591-024-02964-1

[137] Németh T , Nagy G, Pap T. Synovial fibroblasts as potential drug targets in rheumatoid arthritis, where do we stand and where shall we go? Ann Rheum Dis 2022;81:1055–64. 35715191 10.1136/annrheumdis-2021-222021 PMC9279838

